# Key Features of Culturally Inclusive, -Affirming and Contextually Relevant Mental Health Care and Healing Practices with Black Canadians: A Scoping Review

**DOI:** 10.3390/ijerph22091316

**Published:** 2025-08-23

**Authors:** Sophie Yohani, Chloe Devereux

**Affiliations:** Faculty of Education, University of Alberta, Edmonton, T6G 2G5 AB, Canada; cdevereu@ualberta.ca

**Keywords:** mental health, Black Canadians, culture, healing, anti-Black racism, social justice

## Abstract

Black Canadians are one of the fastest-growing groups in Canada, with 59% of this population comprising immigrants. Ongoing systemic racism and discrimination have serious consequences for the mental health of Black Canadians. While research and policy efforts to address the mental health needs of this population are ongoing, a greater understanding of the healing practices relevant to this diverse population is needed. This scoping review synthesized and discussed key features of culturally inclusive, affirming, and contextually relevant approaches and practices for mental health care and healing with Black Canadians, as well as identified limitations and gaps in the current research. This study followed the PRISMA guidelines for scoping reviews and conducted a search in PsycINFO, MEDLINE, Embase, SocINDEX, CINAHL, Sociological Abstracts, and Global Health in October 2023. A total of 34 articles met the inclusion criteria. The review identified that most studies were conducted in one Canadian province (i.e., Ontario) and involved diverse perspectives, including service users and providers. The thematic review of articles revealed limited research regarding specific interventions, but identified many commonly reported features of culturally and contextually relevant approaches to mental health care and healing for Black Canadians that broaden the scope of mental health care beyond Euro-Western clinical models, including taking a holistic and empowerment-based approach, engaging in culturally affirming care, a social justice approach, community-centred and collaborative healing, and the necessity of practitioner education. Recommendations for practice, policy, education, and research are provided to support more inclusive and responsive mental health care systems for Black Canadians.

## 1. Introduction

Black Canadians account for 4.3% of the total Canadian population and are one of the fastest growing groups, having increased from 1.2 million to 1.6 million between 2016 and 2021, and are expected to reach 3 million by 2041 [1]. While recent growth is attributed to immigration, with 59.1% having been born outside of Canada, Black Canadians have lived and contributed to the growth and development of Canada since the 16th Century. Despite their long-term and growing presence in Canada, many Black Canadians live in poverty, have low educational attainment, and are overrepresented in the criminal justice and child welfare systems (see [2]). In fact, the 2017 report of the United Nations (UN) Working Group of Experts on People of African Descent (UNPAD) on its mission to Canada highlighted the cumulative impact of systemic racism and discrimination faced by Black Canadians as the main factor preventing their rights to economic, social, and cultural liberties [3] (p. 7). This report echoed the long-raised concerns by researchers of Black mental health (e.g., [4]), including recent research establishing links between experiences of everyday racism, microaggressions, and anti-Black racism and anxiety, depression, and trauma responses amongst Black Canadians (see [5,6,7]). Since the UN’s report and Canada’s endorsement of UNPAD in 2018, there have been a number of national initiatives to address the mental health and well-being of Black Canadians, including funding for research and mental health projects in Black communities (e.g., see [8]). While these projects have begun to address the critical knowledge and service needs of Black communities, there remains a dearth of published research specifically focused on mental health interventions with Black Canadians. Relatedly, there is a need for more information on culturally informed practices that address anti-Black racism and the mental health care needs of Black Canadians to support ongoing institutional and community-level efforts and inform practitioner education. Given these considerations, the goal of this review was to systematically examine current research on culturally and contextually informed mental health care and healing practices for Black individuals and communities in Canada.

We intentionally use the phrase mental health care and healing practices to capture the broad range of interventions reflected in the literature. We acknowledge that the term ‘healing’ carries diverse meanings across biomedical, complementary, and alternative health paradigms, and can be viewed as a state, a process, an outcome, or an intervention [9,10]. In this review, we conceptualize healing primarily as a process or intervention, referring to the actions or practices perceived as beneficial to the mental health of Black Canadians. This use of inclusive and culturally attuned language is especially important, as cultural beliefs and values shape how individuals conceptualize wellness and the means through which they seek support and treatment. This framing aligns with Wampold’s view that psychotherapy is just one of many healing practices across cultures, and that its effectiveness depends on the alignment between client and clinician’s expectations [11]. The therapeutic or healing process has particular relevance here:

“adapting psychotherapy for various cultural groups may be insufficient if psychotherapy is not considered a viable healing practice by the patient. Psychological services that take culture into account may find that psychotherapy is not a valid intervention for some patients of some cultural groups. Whatever the case, the therapist or healer must listen carefully to discern not only what is the problem, how it is conceptualized, and what the goals of the intervention are, but also how the patient wants to heal” [11] (p. 82)

The voices of researchers, practitioners, clients, and community members within the reviewed studies illuminate the diverse ways in which Black Canadians envision healing. These perspectives broaden the scope of mental health care beyond Euro-Western clinical approaches to include community-based supports, culture-specific practices, and justice-oriented activities. Through this synthesis of key contextually and culturally informed approaches, and an identification of research limitations and gaps, this review offers critical insights to guide researchers, educators, practitioners, and policymakers in supporting more inclusive and responsive mental health care systems for Black Canadians.

## 2. Materials and Methods

### 2.1. Design

We conducted a scoping review following the PRISMA-ScR extension for scoping reviews [12] and Arksey and O’Malley’s [13] approach with additional guidance from Levac and colleagues [14] to systematically review current research examining mental health care and healing approaches and practices for Black individuals and communities in Canada. Our protocol for the review is registered with OSF (registration available at: osf.io/57smz). We followed a five-step approach, which involved (1) identifying a research question; (2) identifying relevant studies; (3) selecting studies; (4) charting the data; and (5) collating, summarizing, and reporting the results. The review was guided by the following questions:

1. What approaches to mental health care and healing have been used with Black Canadian populations?
2. What are recommended practices for mental health care and healing with Black Canadians that are context and culturally informed?
3. What gaps exist in the current knowledge of context and culturally informed mental health care and healing approaches with Black Canadians?

### 2.2. Search

**Figure 1:**
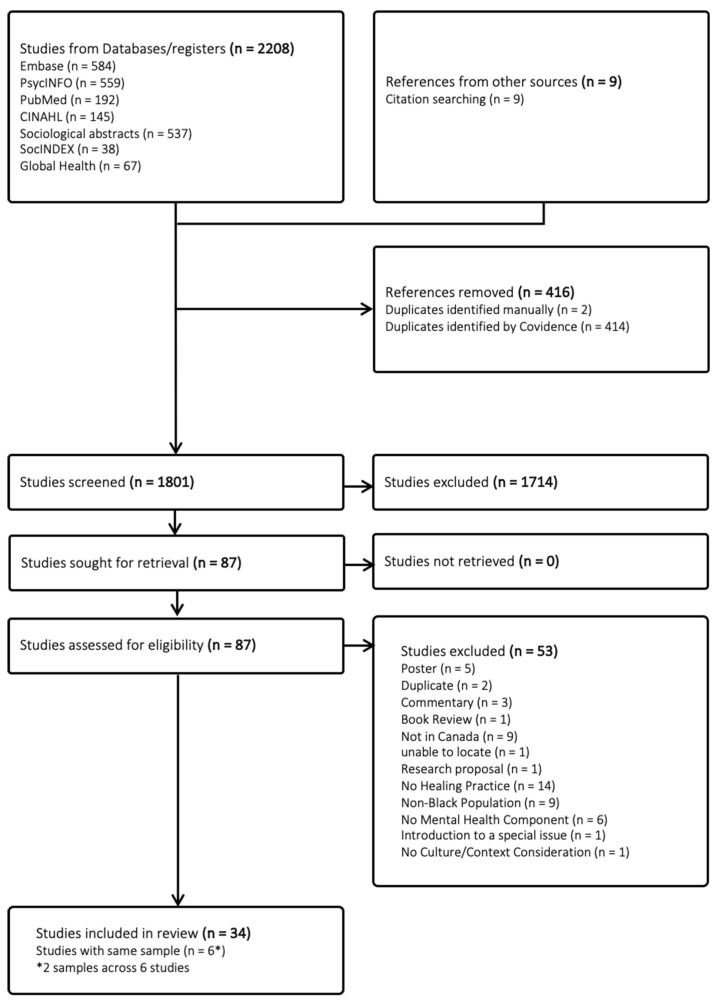
PRISMA flow diagram of the search process. Note. PRISMA = Preferred Reporting Items for Systematic Reviews and Meta-Analyses.

Articles were identified through a systematic search in relevant electronic databases (PsycINFO, MEDLINE [PubMed version], Embase, SocINDEX, CINAHL, Sociological Abstracts, and Global Health) (see Figure 1). All selected databases were searched from inception to 2 October 2023, without date limitations. Through consultation with a health research librarian, we developed a search strategy that involved a combination of natural language keywords and subject headings from the relevant search thesauri for each database, as well as Boolean search operators and truncation (i.e., AND, OR, NOT, *). Our search included five concepts:

1. African, Caribbean, and Black populations (e.g., Africa, Caribbean, Black, names of all African and Caribbean countries, African-centred);
2. Canadian context (e.g., Canad*, names of all Canadian provinces, and 100 most populated cities);
3. mental health intervention approach (e.g., psychotherapy, counsel*, traditional heal*);
4. mental health (e.g., psych*, “mental health”, stress);
5. cultural considerations (e.g., culturally sensitive, cultural adaptation, culture safe, culture informed, local adapt*).

The full search strategy can be seen in Appendix A. The results from the search were imported into Covidence.org. Additionally, we screened references cited in included articles to identify any additional relevant articles.

### 2.3. Screening

The two authors independently screened the titles and abstracts of the retrieved articles, followed by the full-text articles. Following each stage, we met to resolve any conflicts from the screening processes. The inclusion criteria were as follows: (1) includes African, Caribbean, and/or Black populations; (2) involves a Canadian setting; (3) describes a mental health healing practice; and (4) discusses considerations made regarding culture and/or context. The exclusion criteria were the absence of any of the above inclusion criteria (i.e., Non-African, -Caribbean, -and/or -Black population; non-Canadian setting; no mental health healing approach or practices; and no mention of cultural or contextual considerations), studies with pharmacological interventions, as well as articles having insufficient content to review (i.e., poster, abstract, commentary, book review, proposal). In our review, we included articles with mixed group studies if the Black population was clearly defined (i.e., the article provided demographic information regarding race and ethnicity) and data was disaggregated so that findings were clear. 

### 2.4. Data Collection

We extracted relevant data from the included articles using a standardized table with the following categories: study aim, geographic location, name of Black population and country of origin, description of healing approach, target of healing approach, target gender of healing approach, target age of healing approach, number of participants, type of participant, participant gender, participant age, study design/approach, theoretical framework of methodology, theoretical framework of intervention, method of data collection, method of data analysis, cultural and contextual considerations in data collection, analysis, and reporting, cultural and contextual considerations in the healing approach, methodological limitations/challenges/issues, suggestions/recommendations for future research, healing approach limitations/challenges/issues, suggestions/recommendations for practice, results/findings, and other recommendations. The extracted data was then reviewed, organized into themes, and summarized narratively. Throughout the analysis process, we worked closely and engaged in reflective discussions.

## 3. Results

### 3.1. Article Characteristics

The article characteristics can be found in Appendix B. A total of 34 articles were included; however, we are reporting on 30 articles because six of the original articles shared two samples. The majority of reviewed articles were from Ontario (*n* = 19), followed by Alberta (*n* = 6), Quebec (*n* = 3), Manitoba (*n* = 1), and Nova Scotia (*n* = 1); three articles included a national perspective. Nineteen articles were qualitative, three were quantitative, four were mixed-methods, three were case examples, and one was a commentary. The number of participants included in the empirical articles ranged from 1 to 1485.

Participant roles included: community members (*n* = 12), clients (*n* = 7), service providers (*n* = 1), community members and clients (*n* = 2), community members, service providers, family members of clients, community leaders (*n* = 2), service providers, policy makers, community leaders (*n* = 1), community members, clients, service providers (*n* = 2), community members, clients, service providers, policy makers (*n* = 1), Traditional healers (*n* = 1), not applicable (*n* = 1). Participant genders included: women (*n* = 8), men (*n* = 2), combined women and men (*n* = 15), combined women, men, and gender diverse (*n* = 3), not specified (*n* = 1), and not applicable (*n* = 1). The participants reflected the following age groups: adult (*n* = 15), adult and older adult (*n* = 8), youth (*n* = 1), youth and adult (*n* = 2), youth, adult, older adult (*n* = 2), child and adult (*n* = 1), and not applicable (*n* = 1).

Gender groups included in the healing approaches were combined men and women (*n* = 11), combined genders including gender diverse (*n* = 2), women only (*n* = 8), men only (*n* = 1), and eight were unspecified.

The healing approaches reflected a variety of age groups, including adults (*n* = 16), adults and older adults (*n* = 1), youth (*n* = 2), children and youth combined (*n* = 1), youth and adult combined (*n* = 2), all ages (*n* = 2), and six were unspecified. The focus of interventions included general mental health (*n* = 6), general mental health for children and youth (*n* = 3), general mental health for recently postpartum newcomer moms (*n* = 1), general mental health for young moms (*n* = 1), general mental health for refugees (*n* = 1), reintegration following incarceration for youth/young adults (*n* = 1), mental illness/mental health problems (i.e., psychosis) (*n* = 5), impacts of racism/racial stress (*n* = 4), cultural identity (*n* = 1), challenges with migration/integration/settlement (*n* = 1), social determinants of health (SDOH) for 2SLGBTQIA+ (*n* = 1), social support for refugees (*n* = 3), childhood sexual abuse recovery for men (*n* = 1), spirit injury for women (*n* = 1), and sexualized violence for refugees (*n* = 1).

### 3.2. Methodological Characteristics

We examined methodological limitations and challenges reported in the included articles and noticed several recurring considerations highlighted by the authors. More than half of the studies indicated non-generalizability as a main limitation, citing small sample sizes and limitations in the geographic location of the study participants (i.e., single Canadian province), the demographic characteristics of the participants (e.g., age, gender, sexuality, immigration status, etc.), and ethnicity of the participants. Article authors also noted challenges with recruitment (e.g., constraints related to COVID, convenience sampling), issues with data collection methods (e.g., interview challenges, limitations of measurement tools), and limitations of study designs (e.g., correlational and cross-sectional data, repeated measurement, subjectivity). While most studies reported on methodological limitations, several (*n* = 8) did not.

### 3.3. Contextually and Culturally Informed Mental Health Care and Healing Approaches and Practices

We identified four themes representing key approaches to mental health care and healing employed and identified in research with diverse Black Canadian communities (Table 1). A fifth theme speaks to the knowledge, skills, awareness, and education needed to integrate the identified approaches. We also present sub-themes describing the recommended practices identified by article study participants and researchers within each theme. It is important to note the overlap between themes; for example, recognizing the role or influence of family in healing can reflect both a culturally safe and holistic approach. The themes are described in the following section.

#### 3.3.1. Culture Affirming Care (Cultural Safety, Humility and Competency)

This theme speaks to practices that affirm the cultures of Black Canadians in the context of mental health care and healing. Culture may involve values, beliefs, norms, rituals, and language, and common experiences and histories that bind people together [15]. Given the diversity within the Black Canadian population, clients will have different culture-rooted views, presentations, and coping mechanisms related to mental health, requiring the practice of cultural safety, humility, and competency. The sub-themes outline specific practices that highlight these key points. A critical practice, according to the articles, involved practitioners being aware of and open to the different explanatory models of psychological problems/philosophical ideologies of clients and how culture influences ways of communicating distress and ways of coping [16,17,18,19]. Practitioners must acknowledge the diversity within the Black Canadian population by actively assessing each client’s identity and cultural background, and by avoiding homogenous or ‘one-size-fits-all’ approaches to service delivery [16,18,20]. Culturally affirming approaches require practitioners to continually develop cultural competence by ensuring cultural safety and practice from a stance of cultural humility [18,19,20,21,22,23,24].

Some of the common practices that were mentioned in our review included traditional healing, attending to spirituality and/or religion, and recognition of the importance of family or community.

Culture-Specific and Traditional Healing Practices. Many of the article authors discussed the integration of client-informed approaches for mental health healing, highlighting the importance of attending to unique client factors in the context of their culture(s) [16,18,19,20,23,25,26,27,28,29,30,31,32,33,34,35,36]. This is also the basis for an intersectional approach (see section below). Participants and researchers brought to light various cultural and traditional approaches to mental health care and healing, representing a wide range of practices across diverse Black Canadian cultures and populations [19,23,25,26,27,28,29,30,31,32,33,35]. For example, Sutherland [19] described Caribbean traditional healing, including culture-aligned readings, cleansing (baths and herbal medicines), prayer and meditation (emotional and physical grounding and connection to spirits), and healing ceremonies (a range of rituals, chants, dances, trances, and offerings provided by healers). Moodley & Bertrand [30] conducted interviews with traditional African Caribbean healers and found that the healers considered the etiology of clients’ mental health difficulties, noting that for some, there were spiritual explanations, and thus they found support for incorporating spiritual healing. Other ways of enacting culturally affirming care include providing services in the clients’ first language, services that employ an African-centred framework (i.e., framework which reflects perspectives, experiences, values, worldviews and knowledge systems that originate from African cultures and people of African descent such as the principles of Kwanzaa), or services led by like-ethnic community members [27,28,29,31,32,33,35]. Some authors recommended an integrative approach and/or cross-cultural framework, where traditional cultural and Western practices could be combined in a manner that is sensitive to contextual nuances for each client [20,30,36].

Recognition and Incorporation of Spirituality and/or Religion. Another culturally informed practice is recognizing the importance of spirituality and religion in many African, Caribbean, and Black cultures. Participants and researchers across several articles suggested spiritual and religious approaches to healing [16,19,21,22,24,25,26,30,31,32,33,35,37,38,39,40,41,42]. Religious and spiritual approaches involved prayer, attending religious centres such as churches and mosques, reading scripture, and other spiritual rituals [16,19,22,31,32,33,35,37,38,42]. In their study with Jamaican women who immigrated to Canada, Dixon and colleagues [22] indicated that practitioners “need to be humble, curious, open, spiritually sensitive, and non-judgmental in their work” (p. 2196) especially when working with clients with faith practices, as spirituality and faith practices are a critical aspect of culture for this population. Accordingly, practitioners should strive to: develop knowledge regarding various religions and spiritualities practiced by their clients; be comfortable engaging in dialogue regarding religion, faith, and spirituality with clients; have resources and referrals for religious support at the request of clients; and acknowledge the potential resource of religion and spirituality for various clients [38]. Importantly, practitioners should let clients guide the inclusion of spirituality and religion, and be sensitive to the diverse relationships with religion and spirituality that Black Canadian community members have [16,19,22,24,38]. Two articles further recommended that mental health practitioners collaborate with churches to raise awareness of mental health and de-stigmatize accessing other supports in addition to faith-based supports [16,38].

Option for Black Mental Health Service Providers. Enhancing cultural safety in mental health care for Black Canadians includes ensuring the option to work with Black mental health professionals. Several articles noted the need for more Black service providers [17,24,36,39,43]. Participants in articles articulated the importance of having Black mental health service providers as a way of increasing accessibility and acceptability of mental health interventions [17,36,39,43]. As an example, participants from two African refugee groups who were new parents indicated their preference for same-ethnicity service providers; they wished for service providers who were knowledgeable about traditional postpartum practices, community elders to support with individual and marital issues and childcare, and language interpreters in medical and community settings to provide culturally sensitive support [31,32,33]. While cultural differences exist throughout the Black population in Canada, having providers with lived experience of being Black in Canada may decrease the emotional labour of Black clients in interventions (i.e., from needing to explain their experiences with anti-Black racism, etc.) and increase a sense of feeling understood and accepted [36].

Address Mental Health Stigma. Researchers in reviewed articles indicated a need to provide psychoeducation for clients and community members to reduce mental health stigma [16,19,20,38,40,41]. Education that builds upon the specific community’s understanding and cultural knowledge may help people to understand symptoms of mental health problems, provide appropriate support options, educate on the impacts of trauma in culturally congruent manners, educate in a way that facilitates resilience and adaptation, and involve community leaders (such as faith leaders) [16,19,20,38,40,41]. By attending to stigma, practitioners increase their clients’ sense of safety, address specific concerns, and collaborate to ensure appropriate fit of services.

Recognition and Incorporation of Family and Community. A component of culturally informed and affirming care is the recognition of relationality in Black communities. This means moving beyond the focus on the individual client to recognizing the role of family and community in supporting healing and well-being. For example, cultural rituals such as the coffee ritual practiced by Ethiopian women described in King and colleagues’ study [26] and social gatherings with like-ethnic family and friends, were noted to be important coping resources for mental health across many studies [21,25,31,32,33,35,44]. Family- and community-focused approaches are discussed further in other themes/sub-themes.

#### 3.3.2. Holistic and Empowerment-Based Approach to Mental Health

A major finding in our review is the consistent message that mental health care and healing approaches with Black Canadians should be attuned to the “whole” person, including physical and psychological self, spirit, and social ties. This reflects an approach that seeks to work with clients in a way that is responsive to their individual needs and embodies an openness to step outside of Western and Eurocentric approaches. This might mean different approaches to trauma interventions, including family members in interventions, and including spirituality and/or religion, as well as working from a strength-based and resilience-focused approach, rather than focusing on challenges, as summarized in the following sub-themes.

Use Holistic and Flexible Approaches. Mental health care and healing approaches with Black Canadians often attend to the whole person (mind–body–spirit) and the interconnectedness between person and context [19,21,24,26,30,31,32,33,34,35,42]. When providing holistic support, interventions should also be flexible to adapt to the unique needs of clients [19,24,34]. Holistic and flexible interventions may integrate Western and traditional frameworks of healing, include body-focused approaches, incorporate spirituality, as well as attend to diverse experiences of trauma [19,21,24,30,31,32,33,35,42]. Recognizing spirituality and religion as a potential element of holistic and flexible healing overlaps with providing culture-informed interventions. For example, Baiden & Evans [21] examined perspectives of Black African immigrant women regarding postpartum mental health services, and identified that study participants desired holistic approaches that incorporated diverse interventional modalities, namely “non-medical” intervention options, including combinations of spiritual, social support groups, spousal involvement, as well as Western mental health support. Due to the highly diverse nature of the Black Canadian population, practitioners should strive to intervene in ways that align with clients’ unique circumstances and needs, going beyond typical Western notions of psychotherapy.

Use Collaborative and Client-Centred Individualized Approaches. In order to provide holistic approaches that align with clients, participants and researchers alike recommended the use of a collaborative and individualized stance [17,21,34,35,38,40,42]. Participants shared many personal self-help and coping strategies that they employ in their lives, including boundary setting, journaling, finding balance, meditation and relaxation, engaging in positive self-talk, listening to Black activists, reading books about others who have dealt with racism, gardening, baking, finding relatable external sources of information, solitude, diet regulation, spiritual practices, exercise, puzzles, travelling, reappraising challenges, and selectively talking with family and friends [17,21,35,40,42]. By taking a client-centred approach, these personal healing activities that Black Canadians draw upon can be incorporated intentionally into mental health interventions. Likewise, researchers and participants both noted that when working one-on-one with clients, the practitioner should take a collaborative stance and acknowledge that each client will have diverse beliefs regarding mental health care and healing based on various intersecting factors [27,28,29,34,35,38]. Gopaul-McNicol and colleagues [39] describe a case example with a Haitian client, detailing how the practitioner worked collaboratively to meet the client’s specific individual needs by understanding and respecting the client’s cultural beliefs regarding the involvement of Voodoo in her experiences; encouraging the client to seek spiritual healing; connecting the client with a Haitian psychologist for a re-assessment in her native language; referral to an educational program based on client’s interests; and connecting the client with a Haitian community centre. Involving clients in making intervention choices ensures the interventions used are appropriate and acceptable for each client.

Use Strengths-Based and Resilience-Enhancing Approaches. An aspect of holistic healing involves engaging in mental health care and healing approaches that are strength-based and emphasize building and enhancing resilience [17,19,24,25,26,27,28,29,31,32,33,34,41,45]. While acknowledgement of challenges is an important aspect of mental health care, healing with Black Canadian communities should focus on identifying and developing strength-based coping and resilience [24,26,31,32,33,34]. This can involve connecting clients with culturally based supports that enhance coping with challenges, fostering within ethno-cultural community connections and traditions, and addressing settlement needs in a way that encourages empowerment, agency, and confidence [19,26,27,28,29,31,32,33,45]. Researchers suggested that mental health care approaches should be recovery-oriented, include non-clinical interventions (e.g., recreation-based activities, particularly for children), support families staying together, and seek to redefine how resilience is perceived and experienced in Black communities [17,25,26,31,32,33,41].

Use Trauma-Focused and -Informed Approaches. A few of the reviewed articles recommended that mental health care and healing approaches should be trauma-focused and trauma-informed [19,25,38,45]. One article, specific to Caribbean clients in Canada, stated that practitioners should work from a paradigm that addresses the range and diverse nature of trauma, including social, cultural, political, and historical (e.g., displacement, racism-related, and intergenerational trauma), that clients may be experiencing and ensure that they are considerate of the clients’ explanatory models of trauma [19]. This article also noted that the therapeutic alliance should be emphasized, and interventions should be tailored to fit the needs of clients to ensure safety, trust, choice, and control, and that therapy should address loss and disconnection from history and ancestry [19]. Another article also noted that trauma-informed care should be aware of the diverse faiths of clients [38]. One article noted that intrusive trauma-focused approaches (e.g., Trauma-Focused Cognitive Behavioural Therapy) should initially be avoided when working with African refugee women who have experienced sexualized violence, and that trauma interventions should be resilience-focused and adaptive to the cultural and settlement needs of clients [45].

Use Family-Focused Approaches. Participants from research studies suggested that mental health care approaches should be family-oriented [21,25,31,32,33,35]. For example, work with children and youth should include parents, and maternal care should include spouses and partners [21,25]. Authors also highlighted the importance of addressing the influence and needs of family units by working with family members of clients, with couples, and engaging in outreach to family homes [21,25,31,32,33]. Family-oriented approaches to mental health care and healing may also include cultural and recreational resources and activities for families (e.g., informal talks with elders, summer recreation programs for children) [31,32,33]. Recognition of the transnational nature of migrant families, recognition of ongoing links to families in countries of origin and the diaspora, is critical as these links shape decision making, provide support, and play a role in mental well-being (e.g., both adaptive and limiting) [31,32,33,44].

#### 3.3.3. Social Justice Approach to Mental Health

This theme speaks to the structural, systemic, and socio-political–historical factors that impact the mental health of Black Canadian populations and that should be addressed in mental health care. This means addressing racism and racism-related mental health impacts and recognizing Social Determinants of Mental Health (SDoMH). This also involves addressing intersecting identities, including gender and sexuality, amongst others, and how these contribute further to the marginalization of diverse members within Black populations in Canada. The role of mental health practitioners must importantly include advocacy.

Address Racism and Racism-Related Stress. Participants and researchers alike provided recommendations addressing racism and racism-related stress as a critical component of mental health care and healing [16,17,18,19,23,26,35,36,43]. Participants endorsed diverse strategies to cope with racism, including building resilience and confidence in the face of racism, directly addressing racism, selectively attending to racism, spiritual-centred coping, problem-solving coping, emotional debriefing, collective coping, exchanging information on how to cope with racism with others, and ignoring racism [16,17,18,23,25]. Stakeholders in one study highlighted that there is a need for the humanization of Black Canadians with mental illness, particularly in policing and health care settings [23]. Researchers from studies recommended that practitioners need to be aware of the impacts of racism on mental health (e.g., racism-related stress), as well as the diverse and intersecting contexts that clients are situated in [16,19,43]. Interventions and mental health sites should not only implement anti-racist frameworks and actions, but also directly address racism and discrimination as a part of interventions [19,23,26,35]. For example, Greene [17] insisted that practitioners “validate, acknowledge, and build resilience/resistance in Black individuals” (p. 214). This includes practitioners having an awareness of “issues related to anti-Blackness and white supremacy” in order to facilitate healing and empowerment with clients [17]. Towards addressing the mental health impacts of systemic racism, other institutions, including schools and children’s services, need to have a better understanding of anti-racism, racism, xenophobia, and immigration (i.e., via training; [43]).

Attend to Social Determinants of Health. Both participants and researchers highlighted that having a Social Determinants of Health (SDoH) lens is vital to understanding the mental health of Black Canadians. Several articles noted the importance of addressing education, employment, housing, and financial needs [17,23,25,26,35,41,45]. Attending to SDoH means not only addressing these topics as part of interventions, but also tailoring interventions to decrease barriers to access related to SDoH. Further, SDoH represent areas to be addressed in larger policy and government-level institutions. Particular attention is needed for communities with intersecting identities, including Black 2SLGBTQIA+ community members [46]. Authors described the need for practical supports, such as accessing childcare, securing stable employment, in-home support with parenting and family life, and institutional changes that advance the basic rights of Black Canadians to have equal access to housing, employment, and income [23,26,27,28,29,31,32,33,41,45]. Makwarimba and colleagues [27,28,29] offer an exemplary approach to a group intervention with African immigrants that considered SDoH from an accessibility standpoint. Based on feedback from community members, they situated the group at an in-person location that was familiar to most participants and addressed the technological barriers of online treatments. They also covered the cost of transportation and provided childcare for those with children. The role of social support as a key aspect of SDoH was also notable in this study [31,32,33].

Take an Intersectional Lens. A handful of articles highlighted the importance of working from an intersectional lens [19,20,43,44]. This involves recognizing the intersecting identities and diversity within the Black community in Canada, and understanding how clients who hold historically marginalized social identities, including gender, race, ethnicity, sexual orientation, nationality/migration status, and disability, may face increased marginalization [20,43,44]. Additionally, mental health models should recognize the prevalence of systemic racism, ongoing impacts of colonialism, intergenerational trauma, and prolonged exposure to anti-Black racism and the related impacts on Black peoples globally [19,20,43].

An intersectional perspective amplifies the need noted by researchers in reviewed studies to address and attend to homophobia and gender-based discrimination faced by Black Canadians [38,46]. In the context of mental health service delivery, one article underscored the need for practitioners to account for cultural variation in the acceptance of 2SLGBTQIA+ across different communities in order to more effectively attend to the diverse lived experiences of Black 2SLGBTQIA+ clients [38]. Regarding larger-scale advocacy efforts, these authors noted that there needs to be accountability, conversation, and safety measures for homophobia and transphobia experienced by Black 2SLGBTQIA+ from faith-based groups and individuals [38]. Another article highlighted the urgent need for increased support for 2SLGBTQIA+ African Canadians, including social support (e.g., support groups), improved access to health services, culturally and 2SLGBTQIA+ competent mental health services, and information and resources to realize justice and attend to SDoH [46].

Engage in Advocacy. Mental health care and healing practices with Black Canadians involves multi-level advocacy both within communities and at institutional levels [22,23,31,32,33,34,45,46]. One study summarized this well by stating that practitioners should engage in multi-faceted advocacy, including guiding clients to self-advocate, partnering with clients to advocate together, facilitating group efforts to address injustices, and collaborating with colleagues to address injustice [34]. Ungar [34] recounted a case example of an intervention with an African Canadian youth, where he described how his client experienced racism in his school from peers and wished to be transferred to a different school with primarily Black students. Ungar described advocating for this client by contacting the school and arranging for the youth to be transferred to the school he desired [34]. Advocacy is thus needed in the context of therapeutic interventions, within communities and organizations, in addition to being critical in reforming larger institutions, including the justice system and health care system for more humane and just treatment of Black populations (including refugees and those with severe mental illness, amongst others) [23,31,32,33,45,46]. Several researchers also recommended empowerment within communities, such that communities may engage in activities that address their unique needs and solutions [22,31,32,33].

#### 3.3.4. Community-Centred and Collaborative Healing

Many authors in our review emphasized the importance of community and collaborative healing practices when addressing the mental health of Black Canadians. Community group-based practices can be formal (e.g., group therapy; intervention groups) and informal (e.g., faith institutions, community association groups). This also involves mental health practitioners collaborating with community groups as outlined in the following sub-themes.

Mobilize Informal Community Social Support. A number of authors noted how informal community social supports are important avenues of mental health support [16,17,21,26,31,32,33,35,39,40,41,42,43]. Participants in various studies highlighted common use of informal support from family, friends, and cultural community members within their network for a variety of supports, including emotional support through talking and sharing personal experiences, informational support, and practical support, such as childcare [16,17,26,31,32,33,35,39,40,41,42,43]. Researchers underscored the importance of offering both formal (discussed below) and informal social support groups, such that people sharing similar experiences (whether that be like-ethnicity, culture, sexual and gender orientation, etc.) can engage in peer support [21,27,28,29,45,46]. For example, Yohani & Okeke-Ihejirika [45] noted that work with African refugee women who have experienced sexualized violence should prioritize safe and client-led community re-building and support reestablishment of roles and identities in the resettlement context, including support with parenting. One article stood out from the others by recommending the benefits of community spaces where refugees can interact with members of the mainstream community to facilitate mutual learning and networking [26]. Altogether, social support appears essential for mental well-being by enhancing social connections and social capital.

Use Collective Approaches. Group-based approaches provide formal social support while addressing specific mental health needs [21,26,27,28,29,31,32,33,45,46]. Participants recommended collective approaches to healing that are group-based and formed based on shared identities and/or shared focus [27,28,29,31,32,33,45,46]. For example, groups may comprise within ethno-cultural, same age group, marital status, within language, 2SLGBTQIA+, women who have experienced trauma, and refugees, or may focus on experiences with racism, settlement and integration, employment/finances, marriage/relationships, or parenting [27,28,29,31,32,33,45,46]. Stewart [31,32,33] developed and implemented support groups for refugees from Sudan and Zimbabwe. The support groups targeted parents with young children with topics based on the needs of the community, addressing relevant areas impacting mental health, including parenting across cultures, child discipline, managing finances, and career and education support [31,32,33]. Evaluation of the support groups indicated increased perceived support, decreased loneliness and isolation, increased coping strategies, and decreased parenting stress [31]. Collective supports could be offered together by peer leaders and professional helpers to provide advice based on personal lived experiences and support informed by professional experience and resources [27,28,29]. Groups should be tailored and culturally specific to the needs and preferences of the particular population they are intended for.

Engage in Multidisciplinary and Community Collaboration. Researchers also recommended that, in addition to the formation of collective groups for healing, multidisciplinary and community collaboration may extend mental health supports through outreach and collaboration with communities [20,24,25,30,40]. Specifically, researchers suggested that mental health practitioners reach out to faith leaders and sites of worship to support the integration of mental health literacy and destigmatization of mental health within those institutions [16,38]. Relatedly, they highlighted the need for both practitioners and community leaders to be involved in community-based prevention and capacity building by establishing mental health programs to support specific populations [16,38,40]. Another form of cross-field collaboration was identified by Moodley & Bertrand [30], where they discussed the potential for traditional healers and Western medicine institutions to work together.

#### 3.3.5. Practitioner Education

This last theme encompasses the findings that point to essential knowledge, skills, and awareness that mental health practitioners must develop to work effectively with Black Canadians. Studies frequently noted that practitioners should gain knowledge of the diverse cultures of Black Canadians and the impacts of racism on mental health. Further, in order to situate and interact with Black Canadian clients, practitioners need to engage in critical reflection and consciousness raising to better understand their role and positionality.

Practitioner Knowledge and Skills. Nearly half the articles called for practitioner education and skills development around cultural competence, anti-racism, impacts of racism, and contextual realities of Black Canadian populations (e.g., migration, SDOH, etc.) [16,18,19,20,21,22,23,24,30,35,38,41,43]. There is a need for updates to education within academic and mental health institutions to address and expand beyond Eurocentric and racist practices and beliefs [35,43] to include theories that address the realities of Black Canadians, such as critical race theory. Likewise, training and professional development activities are needed for both Black and non-Black practitioners to learn more about multicultural mental health and different approaches to mental health, such as Africentric psychology, intersectionalities, and health disparities facing Black Canadians [20,24,30]. Learning and competence building regarding religion and spirituality, and the traditional healing practices and beliefs of diverse Black Canadians, is also needed [16,22,30,38,41].

Critical Reflexivity and Consciousness Raising. Authors of articles emphasized the importance of developing personal and socio-political awareness amongst practitioners working with Black Canadians. This involves engaging in self-reflection on one’s positionality, biases, and possible complicity within systems of oppression (i.e., critical reflexivity). This also involves developing a deeper understanding of the broader systemic inequalities that affect Black Canadians in mental health (i.e., consciousness raising). A stance of cultural humility where practitioners remain open to ongoing learning, and engaging in critical reflexivity to identify and examine their own biases and how these biases contribute to barriers experienced by Black Canadian clients in mental health services is needed [19,22,24].

## 4. Discussion

This scoping review responded to our research questions by synthesizing mental health care and healing approaches utilized, recommended practices, as well as gaps in the literature regarding service provision. Below we will highlight the five key approaches we identified, alongside critical reflections regarding recommendations. Finally, we will summarize gaps in knowledge regarding mental health care and healing in the Black Canadian context.

### 4.1. Importance of Culturally Affirming Healing Approaches

The findings of this review highlight the importance of culturally affirming healing approaches to mental health care with Black Canadians, given the history of marginalization of their cultural identities in health care settings. Cultural-affirmation involves the recognition of clients’ explanatory models, beliefs, and healing practices, which are informed by culture in different ways. Calls for practitioners to develop cultural safety and humility in order to assess, conceptualize, and support their clients appropriately resonate with leading cultural psychiatry researchers Kirmayer and Jarvis’ position that systematic attention to culture in clinical practice and systems design is vital for ensuring equity in Canadian mental health care [47]. The findings from this review echo reviews of African-centred mental health interventions in the United States, which also identified such culturally rooted approaches as beneficial to Black populations [48,49].

Culturally affirming approaches to mental health interventions should be considered alongside an awareness of potential tensions with traditional ways of healing, as well as difficulties with accessing Indigenous African traditional healing in the Canadian context. As two studies included in the review found, not only were there diverse and nuanced perspectives regarding integration of traditional and Western healing amongst Black Canadians, but questions arose regarding how integration of different healing systems might occur. We concur with the recommendation to take a cautionary approach to integration and begin by addressing current limitations in the Euro-Western system that act as barriers towards integrating other healing systems [20,30]. Further, due to historical and contemporary instances of expropriation and commercialization of Indigenous and traditional healing by Euro-Western systems and practitioners, integration would also need to be vigilant about the potential for appropriation and Westernization of traditional healing practices and malpractice due to inadequate competency [30]. Lateef et al.’s recommendation to ensure that African-centred interventions are grounded in theory is also critical for the development of evidence-based practices that are beneficial to Black Canadian populations. Practitioners must also be cognizant of the potential limitations of accessing preferred culturally affirming practices for newcomers who lack extended family support in Canada, or for members of smaller Black ethnocultural communities and those living in rural or remote Canadian communities.

### 4.2. Holistic Approach to Mental Health

Whereas Euro-Western mental health interventions typically emphasize individual and biomedical approaches, our review recommends that mental health care and healing interventions for Black Canadians adopt a holistic framework that considers the whole person. This includes attending to spiritual, physical, emotional, and communal well-being, which are dimensions that are rooted in Indigenous African worldviews. Wellness and healing, in this context, are understood as both individual and collective, requiring interventions that reflect this dual focus. A holistic approach also involves recognizing the social, historical, and environmental contexts in which Black Canadians live, across time and place. Ultimately, the incorporation of holistic interventions represents a flexibility, including working collaboratively, from a strength-based and trauma-informed stance, to ensure that the healing interventions and resources that clients are accessing align with their needs.

Given the Euro-Western context of mental health training in Canada, most programs do not take a holistic approach. This can hinder practitioners’ ability to develop relevant understanding and skills and recognize the limitations of their practice. Practitioners are thus encouraged to cultivate cultural humility and reflect on how their approaches may fall short in meeting the needs of Black clients [50]. This includes engaging in collaborative, multidisciplinary practice, by connecting clients to supports that address their holistic needs, such as massage therapy, spiritual and religious communities, and traditional healers, amongst others [51]. These strategies are among those for addressing culture and context in mental health care services reviewed by Kirmayer and Jarvis [47]. They also recommend working with cultural brokers who support translation beyond language to include “contextual and community dimensions of meaning and identify important stressors and sources of support and resilience” (p. 14) [47]. A review of studies with Black faith communities in the US, UK, and South Africa also emphasizes the importance of broadening mental health practice to be more holistic and flexible [52].

### 4.3. Influence of Systemic and Structural Barriers in Mental Health

A major finding of our review centred on considerations of and actions to attend to social, cultural, political, and historical barriers to support Black Canadians’ mental health. While Canadian mental health practices have turned a blind eye to the impacts of racism and other SDoMH, the included studies vocalize the need for practitioners to be knowledgeable about systemic and structural anti-Black racism and racism-related stress and intervene appropriately. Relatedly, practitioners and stakeholders must also consider how anti-Black racism and other SDoH impact access to mental health services (i.e., financial barriers, transportation, language and cultural barriers, etc.). Reviews of mental health care services for Black populations in the U.K. and U.S. identify similar approaches and recommendations to implement interventions which consider SDoH to increase accessibility [52,53,54].

The findings of this review provide further evidence for the expansion of mental health practitioners’ roles to involve an advocacy and a social justice lens. To address this expansion, practitioners must re-examine their role and identify different routes where their skills and position can support the mental health needs of Black populations in Canada. Ongoing research and efforts in this area have produced actionable steps for mental health practitioners to take to expand their role to address the identified structural and systemic gaps in mental health practice. Of note, Ratts et al. [55] developed the Multicultural and Social Justice Counseling Competencies guidelines. Williams and colleagues [50] also provide succinct guidance for mental health practitioners to engage in anti-racist practice. 

### 4.4. Community and Collective Mental Health Support

The research on culturally informed and affirming mental health interventions with Black Canadians emphasizes the need to recognize community as a source of support, healing, and action. Community support exists not only informally through discussions of shared experiences and peer support, but also through formal intervention groups. Collaboration between Western-trained mental health practitioners and other community groups is an avenue to disseminate mental health knowledge, support, and de-stigmatization. Studies in the U.K. and U.S. have reported on the effectiveness of peer-based supports and de-stigmatizing efforts through psychoeducation, as well as the importance of collaborating with communities to develop interventions [52,54,56,57,58].

The primary consideration for practitioners to arise from this review theme is the importance of mental health practitioners stepping out of their siloed therapy practice offices to interact in community spaces. While the Euro-Western view for practitioners to primarily work with individuals or small units (such as couples and families), mental health practitioners have resources and knowledge that could be shared in community to build capacity and collaborate, bridging gaps between often inaccessible services and Black Canadian groups in an outreach capacity, which mirrors findings in other Euro-Western countries [52,54,56]. Efforts with longer-lasting impacts would involve prevention activities rather than common remedial interventions. Importantly, such collaborative activities could address mental health stigma identified in various articles as a notable barrier to the acceptability of accessing support across different settings [20,38,40]. Collaborative training between mental health practitioners and faith-based organizations could bolster a commonly accessed social support for religious Black Canadian persons by promoting and sensitizing faith organizations to mental health concerns and needs, as has been performed in other settings [57,59].

### 4.5. Education and Training of Practitioners in Canada

The studies in this review highlight a critical gap in mental health practitioner training, resulting in the lack of attention to anti-Black racism in care and healing practices with Black individuals and communities. Anti-Black racism not only adversely impacts mental well-being but also intersects with social determinants of health, limiting access to appropriate supports. Without an understanding of racism’s impacts, cultural context, and the limits of Eurocentric psychology, practitioners are ill-equipped to provide culturally safe care. For these reasons, practitioners are encouraged to deepen their understanding of critical race theory, critical multiculturalism, and social justice approaches to mental health practice, while reflecting on their biases and cultivating cultural humility. These theories and practices help explain the causes and lived experiences of racism and expand mental health understanding beyond Euro-Western intervention frameworks.

Practitioner education and training programs form the foundation of mental health practice. The attitudes, values, and pedagogical approaches embedded in these institutions play a critical role in shaping how mental health care is conceptualized and practiced. Despite ongoing challenges, the past decade has seen some important efforts in Canada to integrate more inclusive policies and frameworks into training programs (e.g., [60,61]). For example, the Canadian Code of Ethics for Psychologists now includes a section specifically prohibiting discrimination towards peoples and communities [62] (p. 13). Additionally, the most recent edition of the Canadian Psychological Association’s standards for doctoral program accreditation [63]) includes “Individual, Social, and Cultural Diversity,” which encompasses “awareness and sensitivity” in working with diverse individuals and groups as well as “addressing issues of human rights and social justice in all aspects of training.” (p. 6). While these reflect hopeful steps forward, the widespread actionability of these steps remains to be seen. As noted in five studies in the review, increasing the acceptability of services for Black people in Canada also involves the availability of Black practitioners. In order to address this need, actions are needed on institutional levels to decrease barriers in accessing graduate programs for interested Black students. Barriers including financial, application requirements (often unstated), and discriminatory biases in admissions must be addressed [64]. Further, graduate program content and environment must also be addressed, as within program discrimination, lack of diversity, and ongoing financial barriers have all been noted [50,65,66]. With the implementation of new standards and changes to curriculum and professional development requirements, research to investigate changing attitudes is necessary to assess the degree to which practitioner biases may or may not reflect current attempts to address anti-Black racism within the field [67].

### 4.6. Gaps in the Literature Regarding Mental Health Care and Healing Practices

Our review revealed gaps in the literature regarding mental health care and healing approaches with Black individuals and communities in Canada. The review showed that most studies have been conducted in one Canadian province (i.e., Ontario), with only an additional three studies representing a national sample and perspective. This regional bias may reduce the generalizability of the findings and echoes the findings of a recent review of Sub-Saharan African migrants in Canada that also found an overrepresentation of studies in Ontario [68]. Regarding participants in the reviewed studies, there was a broad representation of community members, clients, and service providers. However, only one study involved traditional healers, and therefore, perspectives from cultural and traditional knowledge holders are significantly underrepresented. Most studies included a mix of women and men, but rarely included gender-diverse participants. This gap, identified by our review, supports calls from other studies to engage in research with Black 2SLGBTQIA+ populations who face additional unique mental health needs [38,46]. Our review also identified significant gaps pertaining to mental health interventions with children, adolescents, seniors, and people with disabilities across the lifespan. Early intervention and prevention efforts for children and families have the potential to curtail downstream mental health challenges, but ongoing challenges with access and acceptability of interventions may limit the benefits of such approaches. As such, there is a critical need for research into culturally affirming interventions for Black Canadian populations to ensure that early interventions and prevention services are tailored to the needs of diverse clientele. The majority of interventions described within the identified articles were for general mental health, as opposed to interventions targeting specific mental illnesses, challenges, or needs. This gap may be a result of our inclusion criteria, which required studies that have a focus on culture or culturally adapted interventions. While this review included 30 relevant studies, the majority did not introduce the use of particular interventions, but rather directed readers to practices and recommendations that can contribute to building more affirming and acceptable interventions for Black people in Canada.

### 4.7. Limitations and Suggested Directions for Future Research

The findings and recommendations included within this review should be considered in light of the existing gaps and limitations of the scoping review. First, in scoping the literature, we can confirm that there is limited published literature identifying best or even promising practices [69] for mental health interventions that are culturally and contextually aware and affirming for Black Canadians. At this point in time, we are in the early phases of having accessible and appropriate mental health interventions available for Black Canadians. The practices, approaches, and considerations outlined herein are viable elements to include when designing and implementing interventions, which could then be evaluated for acceptability and efficacy, illustrating an important next step in research and practice. Given our goal to provide overarching information on a limited study area, we have not engaged in a critical assessment of methods in this review. These remain areas for further research and evaluation. One of the potential challenges for conducting an in-depth qualitative assessment is that mental health practices are addressed across multiple disciplines, reflecting diverse research methodologies. While there is limited published literature, we are aware that there is unpublished ‘grey’ literature available, which we did not include in our review. For example, a team of investigators led by leading Black mental health researcher, Dr. K. McKenzie, created a Culturally Adapted Cognitive Behavioural Therapy manual that has been implemented widely [70]. Initiatives like this one would do well to be replicated in other provinces and with other therapeutic modalities and populations. Finally, the findings within this review are limited to the timeframe of our search. Upcoming projects and newly emerging research exist, with potential relevance to this topic. At the time of this writing, for example, we are aware of emerging research on interventions addressing anti-Black racism [67] and addressing racial trauma using psychedelics [71,72,73]. There is also a plethora of program-based interventions with communities [74] and specific guidelines for addressing mental health with Black Canadians in Ontario [75]. Since the latter was not covered in the scope of this review, we highly recommend a review of the grey literature to synthesize this often overlooked knowledge. Our review did not cover interventions focused on people with neurodevelopmental disorders and studies focused on psychopharmacological treatments. Given the breadth and diverse nature of the literature covered within the current review pertaining to non-pharmacological treatments, a separate scoping review pertaining to culturally and contextually relevant pharmacological interventions with Black Canadians is warranted to provide a clear distinction of topics and a sufficient review of the literature.

Broadly, more research on mental health, mental illness, and mental health interventions with Black communities in Canada is needed, given the relatively limited available literature [19,22,35,40,41,42,44]. Ultimately, this means funding bodies should prioritize Black Canadian populations in research. Regarding sub-populations, those deemed at higher intersecting risk should be prioritized, including refugees, children and youth, survivors of sexual and gender-based violence, and Black Canadians who identify as 2SLGBTQIA+ [27,28,29,31,32,33,38,45,46]. To this list we also add populations that were not adequately covered in our review, including seniors, people with disabilities, and Black Canadian populations living in rural and remote communities that are typically underserved by mental health services. In addition, while we attempted to capture a range of mental health and disorders through our search terms, the included studies mainly include general mental health experiences, rather than specific mental disorders or severe mental illnesses. Additional research regarding mental health care for Black persons experiencing more specific concerns is also needed.

Findings from participants also indicated that research into service delivery is needed. Findings regarding forms of service delivery were limited, though heterogenous. Some participants indicated preferences for in-person support, while others reported preferences for online support for protecting anonymity. There was some acceptance of telephone support for follow-up, as well as some need indicated for information sharing via social media [21,27,28,29,31,32,33]. Given the heterogeneity of findings, more research is needed to clarify preferences and needs for diverse groups within the Black Canadian population to better understand which service delivery mode is best for whom and when.

Finally, given the recommendations and tensions with traditional healing, we also suggest more research on traditional healing amongst Black populations in Canada [19,20,30,35]. Studies examining the effectiveness of traditional healing and other African-centred approaches with Black Canadians must also be grounded in theory.

## 5. Conclusions

In this narrative review, we synthesized published research on mental health care and healing practices with Black Canadians and identified key features of culturally informed and affirming practices that are also contextually relevant to individuals and communities in Canada. In doing so we also identified current limitations and areas for further research. In summary, mental health care and healing with Black Canadians should incorporate a holistic understanding of mental health and cultural-affirming practices that recognize the diversity of explanatory models, beliefs, and practices regarding mental health within Black communities. Interventions need to attend to and address impacts of anti-Black racism in the lives of Black Canadians while recognizing individual and community strengths and community as a source of support, healing, and action. Beyond individual well-being, mental health practitioners and leaders need to attend to socio-cultural, political, and historical factors and intersectionalities to support Black persons’ mental health in Canada. This is an ongoing process of un/learning and building cultural competencies, of critical reflexivity, and ultimately requires an attitude of cultural humility and collaboration.

## Figures and Tables

**Table 1:** Contextually and culturally informed mental health care and healing approaches and practices.

Themes/Approach	Sub-Themes/Recommended Practices
Culture Affirming Care (Cultural Safety, Humility and Competency)	Culture-Specific and Traditional Healing Practices Recognition and Incorporation of Spirituality and/or Religion Option for Black Mental Health Service Providers Address Mental Health Stigma Recognition and Incorporation of Family and Community
Holistic and Empowerment-Based Approach to Mental Health	Use Holistic and Flexible Approaches Use Collaborative and Client-centred Approaches Use Strengths-Based and Resilience-Enhancing Approaches Use Trauma-Focused and Informed Approaches Use Family-Focused Approaches
Social Justice Approach to Mental Health	Address Racism and Racism-Related Stress Attend to Social Determinants of Health Take an Intersectional Lens Engage in Advocacy
Community-Centred and Collaborative Healing	Mobilize Informal Community Social Support Use Collective Approaches Engage in Multidisciplinary and Community Collaboration
Practitioner Education	Practitioner Knowledge and Skills Critical Reflexivity and Consciousness Raising

## Data Availability

Not applicable.

## Abbreviations

The following abbreviations are used in this manuscript:

UN	United Nations
UNPAD	United Nations Working Group of Experts on People of African Descent
PHAC	Public Health Agency of Canada
PRISMA-ScR	Preferred Reporting Items for Systematic reviews and Meta-Analyses- Scoping Review
SDoMH	Social Determinants of Mental Health
SDoH	Social Determinants of Health
2SLGBTQIA+	Two-Spirit, Lesbian, Gay, Bisexual, Transgender, Queer or Questioning, Intersex, Asexual, and other identities.
CPA	Canadian Psychology Association

## Appendix A. Search Strategy

Example Search Strategy for Global Health:

1. (((“African-centered” or “African centered” or “Afrocentric” or “Africentric” or Africa* or “North* Africa*” or “Central Africa*” or “Southern Africa*” or “West* Africa*” or “East* Africa*” or Algeria or Angola or Benin or Botswana or “Burkina Faso” or Burundi or “Cabo Verde” or “Cape Verde” or Cameroon or Central African Republic or Chad or Comoros or Congo or “Cote d’Ivoire” or “Ivory Coast” or Djibouti or Egypt or “Equatorial Guinea” or Eritrea or Eswatini or Ethiopia or Gabon or Gambia or Ghana or Guinea or “Guinea-Bissau” or Kenya or Lesotho or Liberia or Libya or Madagascar or Malawi or Mali or Mauritania or Mauritius or Morocco or Mozambique or Namibia or Niger or Nigeria or Rwanda or “Sao Tome” or Principe or Senegal or Seychelles or “Sierra Leone” or Somalia or “South Africa” or Sudan or Tanzania or Togo or Tunisia or Uganda or Zambia or Zimbabwe) not “african america*”) or (Antigua or Antiguan* or Antilles or Antillean Islands or Aruba or Aruban* or Barbuda or Barbudan* or Bahamas or Bahamian* or Black* or Caribbean or Cuba or Cuban or Curacao or Curacaoan* or Dominica or Dominican* or “Dominican Republic” or Grenada or Grenadian* or Grenadines or Guadeloupe or Guadeloupean* or GuadeloupIan* or Haiti or Haitian or Jamaica* or Martinique or Martiniquais* or Martinican* or Nevis or Nevisian* or “Puerto Rico” or “Puerto Rican*” or “Saint Kitts” or Kittitian* or “Saint Lucia*” or “Saint Vincent” or Vincentian* or “Sint Maarten” or Sint Maartener* or Trinidad* or Trinidadian* or Tobago or Tobagonian* or “Virgin Island*” or “West Indies” or “West Indian*”)).mp. [mp = abstract, title, original title, heading words, cabicodes words] 419,482
2. exp Africa/or black people/or African-Caribbeans/or Caribbean Community/
3. 1 or 2
4. ((Canad* or “British Columbia” or “Colombie Britannique” or Alberta* or Saskatchewan or Manitoba* or Ontario or Quebec or “Nouveau Brunswick” or “New Brunswick” or “Nova Scotia” or “Nouvelle Ecosse” or “Prince Edward Island” or Newfoundland or Labrador or Nunavut or NWT or “Northwest Territories” or Yukon or Nunavik or Inuvialuit) or (Abbotsford or Airdrie or Ajax or Aurora or Barrie or Belleville or Blainville or Brampton or Brantford or Brossard or Burlington or Burnaby or Caledon or Calgary or Cape Breton or Chatham Kent or Chilliwack or Clarington or Coquitlam or Drummondville or Edmonton or Fredericton or Fort McMurray or Gatineau or Granby or Grande Prairie or Sudbury or Guelph or Halton Hills or Iqaluit or Inuvik or Kamloops or Kawartha Lakes or Kelowna or Kingston or Kitchener or Langley or Laval or Lethbridge or Levis or Longueuil or Maple Ridge or Markham or Medicine Hat or Milton or Mirabel or Mississauga or Moncton or Montreal or Nanaimo or New Westminster or Newmarket or Niagara Falls or Norfolk County or North Bay or North Vancouver or Oakville or Oshawa or Ottawa or Peterborough or Pickering or Port Coquitlam or Prince George or Quebec City or Red Deer or Regina or Repentigny or Richmond or Richmond Hill or Saanich or Saguenay or Saint John or Saint-Hyacinthe or Saint-Jean-sur-Richelieu or Saint-Jerome or Sarnia or Saskatoon or Sault Ste Marie or Sherbrooke or St Albert or St Catharines or St John’s or Strathcona County or Surrey or Terrebonne or Thunder Bay or Toronto or Trois-Rivieres or Vancouver or Vaughan or ((Cambridge or Halifax or Hamilton or London or Victoria or Waterloo or Welland or Whitby or Windsor) not (UK or Britain or United Kingdom or England or Australia)) or Whitehorse or Winnipeg or Wood Buffalo or Yellowknife)).mp. [mp = abstract, title, original title, heading words, cabicodes words]
5. exp Canada/
6. 4 or 5
7. (Counsel* or Treatment* or Therap* or Psychotherapy* or intervention or “mental health service*” or “psychosocial support*” or “psychosocial intervention*” or “psychosocial program*” or psychiatry* or “global mental health” or “traditional heal*” or “spiritual heal*” or “religious heal*” or “faith heal*” or “traditional medicine” or “traditional African medicine” or “complementary medicine” or “traditional therap*” or “complementary therap*” or “alternative medicine” or “alternative therap*” or “natural medicine” or “herbal medicine” or “folk medicine” or “holistic medicine” or “traditional healing practice*” or “mind–body” or “faith-based mental healthcare” or “faith based mental health care”).mp. [mp = abstract, title, original title, heading words, cabicodes words]
8. exp psychotherapy/or exp “complementary and alternative medicine”/or exp traditional medicine/or exp traditional health services/or family counselling/or group counselling/or individual counselling/or leisure counselling/or marriage counselling/
9. 7 or 8
10. (Psych* or “mental health” or stress or disorder or mental or mind or psychopatholog* or behavioural or behavioral or emotion*).mp. [mp = abstract, title, original title, heading words, cabicodes words] 649,684
11. exp mental health/or exp mental disorders/or exp mental stress/or exp emotions/
12. 10 or 11
13. (cross-cultura* or “cross cultural*” or multicultural or “culture specific” or “culturally specific” or “cultural specific*” or “cultural identi*” or “cultural background*” or “culturally compared” or “culturally compares” or “culturally compare” or “cultural compar*” or “cultural group*” or “cultural aspect*” or “cultural perspective*” or “cultural factor*” or “culturally adapt*” or “cultural adapt*” or “culturally sensitive” or “cultural sensitivit*” or transcultural or “cultural adjustment*” or “culturally adjust*” or “cultural attunement*” or “culturally attune*” or “cultural tailor*” or “culturally tailor*” or “cultural modification*” or “culturally modif*” or “culturally enhance*” or “cultural enhance*” or “culturally ground*” or “cultural equivalen*” or “culturally equivalen*” or “cultural fit” or “cultural awareness” or “culturally aware” or “cultural knowledge” or “culturally knowledg*” or “cultural understanding*” or “culturally understand*” or “cultural expertise” or “cultural skills” or “culturally informed” or “cultural information” or “culturally safe” or “cultural safe*” or “culturally respon*” or “cultural respon*” or “culturally focus*” or “cultural focus*” or “culturally relevant” or “cultural relevance” or “culturally congruent” or “cultural congruence” or “culturally consistent” or “cultural consistency” or “culturally acceptable” or “cultural acceptability” or “contextual adaptat*” or “contextually adapt*” or “local adapt*” or “locally adapt*” or “cultural consideration*” or “culturally consider*” or “culturally suitab*” or “cultural suitab*” or “culturally adequate” or “cultural adequa*” or “culturally appropriate” or “cultural appropriate*” or “cultural influence*” or “culturally influence” or “culturally tailor*” or ethnocentric or “culturally diverse” or “cultural diversi*” or “linguistically diverse” or “linguistic diversi*” or “cultural differen*” or “culturally differen*” or African-centered or “African centered” or Afrocentric or Africentric or indigen* or “Black psychology” or “ethnically diverse” or “ethnic diversity” or “ethnic consideration*” or “culturally compet*” or “culturally-infused” or “culture-infused” or “cultural infusion” or “culturally-resonant” or “contextually relevant” or “tailor*” or “socio-cultural* adapt*” or “psychosocial context” or “social context” or “community-based approach*” or “co-design” or “participatory approach*” or “holistic” or “contextualiz*” or “African setting*” or “community-based”).mp. [mp = abstract, title, original title, heading words, cabicodes words] 124,748
14. exp indigenous knowledge/or exp cultural values/1201
15. 13 or 14
16. 3 AND 6 AND 9 AND 12 AND 15

## Appendix B. Article Characteristics

**Article**	**Study** **Location**	**Study Design**	**Target of Healing Approach**	**Sample Size**	**Participant Type**	**Participant Gender**	**Participant Age**	**Approach Target Gender**	**Approach Target Age**
Alaazi et al., 2022 [25]	Alberta	Qualitative	General mental health for children and youth.	81	Community members	women, men	adult	not specified	children, youth
Aryee 2011 [37]	Ontario	Qualitative	Psychospiritual healing women living with HIV/AIDS	5	community members, clients	Women	adult	Women	adult
Baiden & Evans 2021 [21]	Ontario	Qualitative	General mental health for postpartum women who immigrated within 5 years.	10	Community members	women	adult	women	adult
Beagan et al., 2012 [16]	Nova Scotia	Mixed-methods	Mental health impacts of racism	50	community members	women	adult, older adult	women	adult
Beausoleil et al., 2017 [76]	Ontario	Quantitative	Reintegration for previously incarcerated youth and young adults.	300	clients	women, men	youth, adult	men, women	youth, adult
Cénat et al., 2023 [77]	Ontario/National	Non-empirical	General mental illness	N/A	N/A	N/A	N/A	not specified	not specified
Dixon & Arthur 2023 [22]	Alberta	Qualitative	Cultural identity	7	community members	women	adult	women	adult
Fante-Coleman et al., 2023a [36]	Ontario	Qualitative	General mental health for youth	128	community members, service providers, family members of clients, community leaders	women, men, trans, gender-fluid	youth, adult, older adult	not specified	youth
Fante-Coleman et al., 2023b [38]	Ontario	Qualitative	General mental health for youth	128	community members, service providers, family members of clients, community leaders	women, men, transgender, gender fluid, third gender	youth, adult, older adult	women, men, 2SLGBTQIA+	youth
Goddard-Durant et al., 2023 [43]	Ontario	Qualitative	General mental health for young mothers	13	Community members	women	youth, adult	women	youth, adult
Gopaul-McNicole et al., 1998 [39]	National	Case example	General mental health	1	client	women	adult	not specified	not specified
Greene 2022 [17]	Ontario	Qualitative	Mental health impacts of racism	10	Community members	women, men	adult, older adult	women, men	adult
Issack 2015 [40]	Ontario	Qualitative	General mental health	8	community members	women, men	adult	women, men	adult
Jarvis et al., 2005 [78]	Quebec	Quantitative	Psychological distress	1485	community members	women, men	adult, older adult	women, men	adult
Joseph & Kuo 2009 [18]	Ontario	Quantitative	Mental health impacts of racism	190	community members	women, men	adult, older adult	women, men	adult, older adult
King et al., 2016 [26]	Manitoba	Qualitative	Psychosocial well-being	15	community members	women, men	adult	women, men	adult
King et al., 2022 [23]	Alberta	Mixed-methods	General mental health for refugees	174	service providers, policy makers, community leaders	not specified	adult	not specified	not specified
Logie et al., 2016 [46]	Ontario	Qualitative	Social determinants of health for 2SLGBTQIA+	29	clients	women, men, transgender women	adult	women, men, 2SLGBTQIA+	children, youth, adult, older adult
Makwarimba et al., 2013/Stewart et al., 2011/Stewart et al., 2012 [27,28,29]	Ontario and Alberta	Mixed-methods	Social support for refugees	51–68	community members, clients service providers	women, men	adult	women, men	adult
Moodley & Bertrand 2011 [30]	Ontario	Qualitative	General mental health	5	traditional healers	women, men	adult	women, men	child, youth, adult, older adult
Myrie 2021 [24]	National	Qualitative	Childhood sexual abuse recovery for men	6	community members	Men	adult	Men	Adult
Osazuwa & Moodley 2023 [20]	Ontario	Qualitative	General mental health	10	community members, clients	women, men	adult	not specified	not specified
Stewart et al., 2017/Stewart et al., 2018/Stewart et al., 2015 [31,32,33]	Alberta and Ontario	Mixed-methods	Social support for refugee parents	67–72	community members, clients, service providers, policy makers	women, men	adult, older adult	women, men	adult
Sutherland 2017 [19]	Ontario	Qualitative	Psychological distress/mental health problems	10	clients	women, men	adult, older adult	women, men	adult
Turner 1991 [44]	Quebec	Case example	Challenges with migration/settlement/integration	1	clients	women, men	adult, child	not specified	not specified
Ungar 2010 [34]	Ontario	Case example	General mental health; impacts of racism	1	client	men	youth	none specified	not specified
Waldron & Gayle 2002 [35]	Ontario	Qualitative	General mental health/mental illness	10	service providers, community members, clients	women, men	adult, older adult	women	adult
Whitley 2016 [41]	Quebec	Qualitative	Mental illness	47	clients	women, men	adult, older adult	women, men	adult
Yohani & Okeke-Ihejirika 2018 [45]	Alberta	Qualitative	Mental health impacts of sexualized violence	6	service providers	women	adult	women	adult
Ziral 2009 [42]	Ontario	Qualitative	Spiritual injuries for women	15	community members	Women	adult	Women	adult
